# Ocular Insights: Exploring Uveitis as a Manifestation of Celiac Disease

**DOI:** 10.7759/cureus.86736

**Published:** 2025-06-25

**Authors:** Javaria Saleem, Abdullah Haroon, Nauman Hashmani, Sharif Hashmani

**Affiliations:** 1 Department of Ophthalmology and Visual Sciences, Hashmanis Group of Hospitals, Karachi, PAK; 2 Department of Medicine, Ziauddin Medical College, Karachi, PAK

**Keywords:** autoimmune enteropathy, celiac disease, immunology, malnutrition, ophthalmology, paediatrics, panuveitis, rheumatology

## Abstract

Celiac disease (CD) is an autoimmune condition that mainly affects the small intestine and often leads to issues with nutrient absorption. People with CD commonly experience symptoms like diarrhea, bloating, stomach pain, fatty stools, unintended weight loss, and low iron levels. In addition to its intestinal manifestations, CD is also associated with a range of extraintestinal features and comorbid conditions. Although uveitis is a rare manifestation of CD, we report, to our knowledge, the first documented case from Pakistan of posterior uveitis secondary to CD in an 11-year-old girl. The patient presented to the OPD with complaints of a gradual, painless loss of vision in both eyes over a four-month period. Notably, she exhibited no associated ocular, GI, or malabsorptive symptoms. On examination, her best-corrected visual acuity was 6/24 in the right eye and hand movement only in the left eye. Ocular findings included bilateral +1 anterior chamber cells without keratic precipitates, posterior synechiae, or iris nodules. The anterior segment inflammation was accompanied by bilateral vitritis, vasculitis, choroiditis, and optic disc atrophy. A multidisciplinary team was assembled to rule out other potential causes of uveitis and to develop a treatment plan. Despite oral and topical corticosteroids, there was no significant control of intraocular inflammation. Immunosuppressive therapy was initiated, but disease progression continued, ultimately resulting in complete vision loss. Typically, children with CD who present with classic symptoms, such as bloating and diarrhea, are promptly diagnosed and treated. However, patients without these hallmark intestinal symptoms may remain undiagnosed, potentially leading to irreversible complications like severe vision loss. This case, along with previous reports in the literature, highlights the importance of considering CD in the differential diagnosis of uveitis, even in the absence of GI symptoms, particularly in patients who are unresponsive to standard steroid therapy and after exclusion of other known causes of uveitis.

## Introduction

Celiac disease (CD) is an autoimmune enteropathy characterized by villous atrophy in the small intestine, leading to malabsorption. It typically presents with malabsorptive symptoms such as diarrhea, abdominal distension, abdominal pain, steatorrhea, weight loss, and anemia [1,2]. The condition is triggered by the ingestion of gluten-containing foods [1], and its primary treatment is a strict gluten-free diet, which generally results in symptom resolution [1]. The manifestations of CD arise from both autoimmune mechanisms and malabsorption [1].

Extraintestinal manifestations of CD include weight loss, osteoporosis, short stature, dermatitis herpetiformis, and infertility [1]. Additionally, the disease is believed to be associated with other conditions such as cutaneous vasculitis, type 1 diabetes mellitus, pericarditis, chronic liver diseases, lactose intolerance, Hashimoto thyroiditis, glomerulonephritis, T-large granular lymphocytic leukemia, inflammatory bowel disease, polyarthritis, and sarcoidosis [2,3], likely due to shared genetic susceptibility involving the HLA-DQ2 and HLA-DQ8 regions [1].

Ocular manifestations of CD are rare but have been reported and include dry eyes, keratoconjunctivitis, retinitis pigmentosa, progressive external ophthalmoplegia, orbital myositis, nyctalopia, cataract, central retinal vein occlusion, and uveitis [1-4]. The incidence of uveitis in CD is seldom documented, and to the best of our knowledge, this is the first case report from Pakistan describing posterior uveitis secondary to CD in a young girl.

## Case presentation

An 11-year-old female child, accompanied by her parents, presented to our eye OPD with a complaint of gradual, painless loss of vision in both eyes for the past four months. The child reported difficulty seeing her surroundings, and her parents were concerned about her well-being. She had been diagnosed with CD at the age of five and, according to her parents, had been on a gluten-free diet (GFD) since her diagnosis.

In August 2023, the parents began noticing changes in the child’s handwriting, followed by a decline in her academic performance, which prompted concerns about her vision. The child and her parents denied any history of ocular pain, photophobia, flashes, floaters, redness, watering, joint pain, cough, respiratory issues, or genital ulcers.

At her initial ophthalmologic evaluation in August 2023, her best-corrected visual acuity was 6/24 in the right eye and hand movement in the left eye. Slit-lamp examination revealed a clear cornea with no keratic precipitates (KPs), +1 cells in the anterior chamber (AC), and no posterior synechiae or iris nodules in either eye. Both eyes showed early posterior subcapsular cataract (PSC), abnormal and dull retroillumination, +1 vitreous cells, vascular sheathing, and dull-appearing maculae. Based on these clinical findings, a diagnosis of posterior uveitis with spillover anterior uveitis was made, and a uveitis workup was initiated.

Four months later, in December 2023, the patient presented to our clinic for a second opinion. At that time, unaided distance visual acuity was counting fingers at 3/60 in the right eye (OD) with no improvement upon correction and no light perception in the left eye (OS). Intraocular pressures were within normal limits in both eyes. Slit-lamp examination showed normal lids and adnexa. The conjunctiva appeared quiet; the right cornea remained clear, while the left cornea had a small macular opacity in the superficial stroma. No KPs were observed. Both eyes had Van Herrick grade 3 angles, with +1 cells in OD and +2 cells in OS.

**Figure 1:**
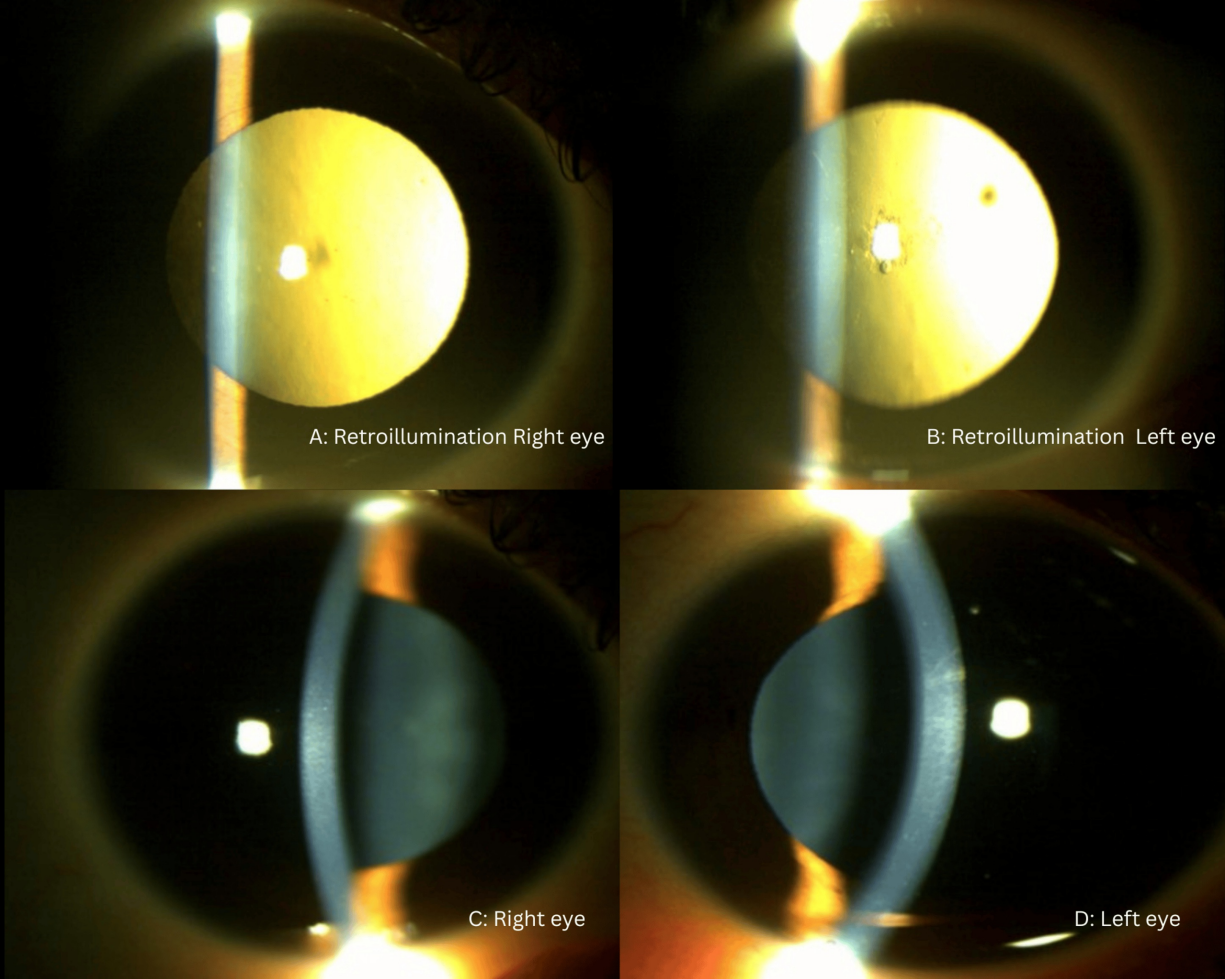
Anterior segment slit-lamp photographs of both eyes (A, B) Dull retroillumination with early PSC in both eyes, more pronounced in the left eye than the right. (C, D) Clear cornea with no evidence of KPs or iris nodules. KP, keratic precipitate; PSC, posterior subcapsular cataract

Pupils were pharmacologically dilated, with no evidence of synechiae. The iris appeared normal and without nodules. Bilateral pigmentary dusting on the anterior lens surface and grade 2 PSCs were noted (Figure 1). There were no signs of xerophthalmia.

**Figure 2:**
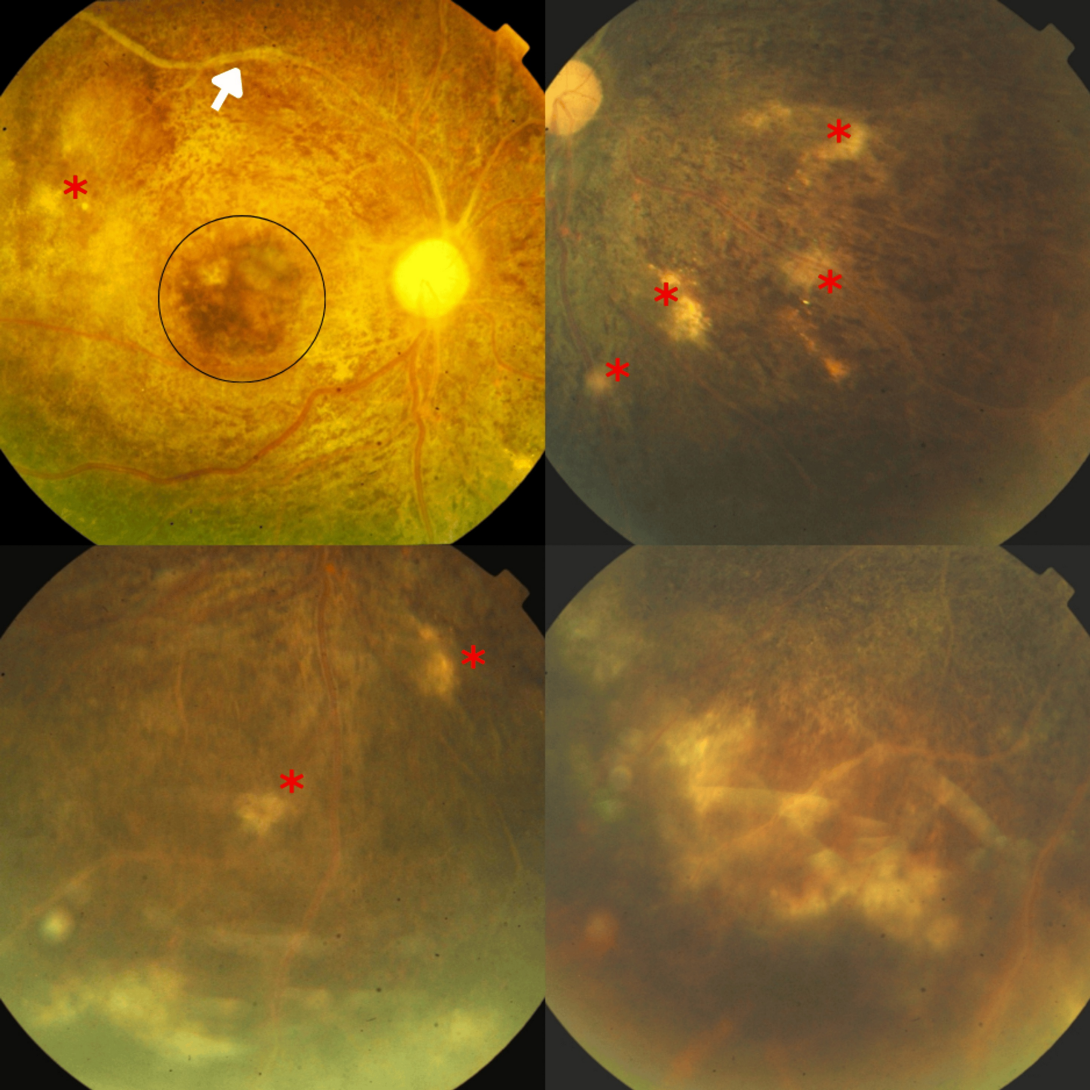
Fundus photographs of the right eye demonstrating a pale, atrophic optic disc with sclerosed vessels in the superotemporal arcade and a hyperpigmented patch at the macula The retinal vasculature is attenuated with signs of perivascular sheathing. Multiple ghost vessels are visible in the peripheral retina, along with mottling, generalized retinal atrophy, and several active choroidal lesions. The black circle marks the hyperpigmented patch on the macula. The white arrow marks the sclerosed vessels in the superotemporal arcade. The asterisk marks the choroidal lesions.

**Figure 3:**
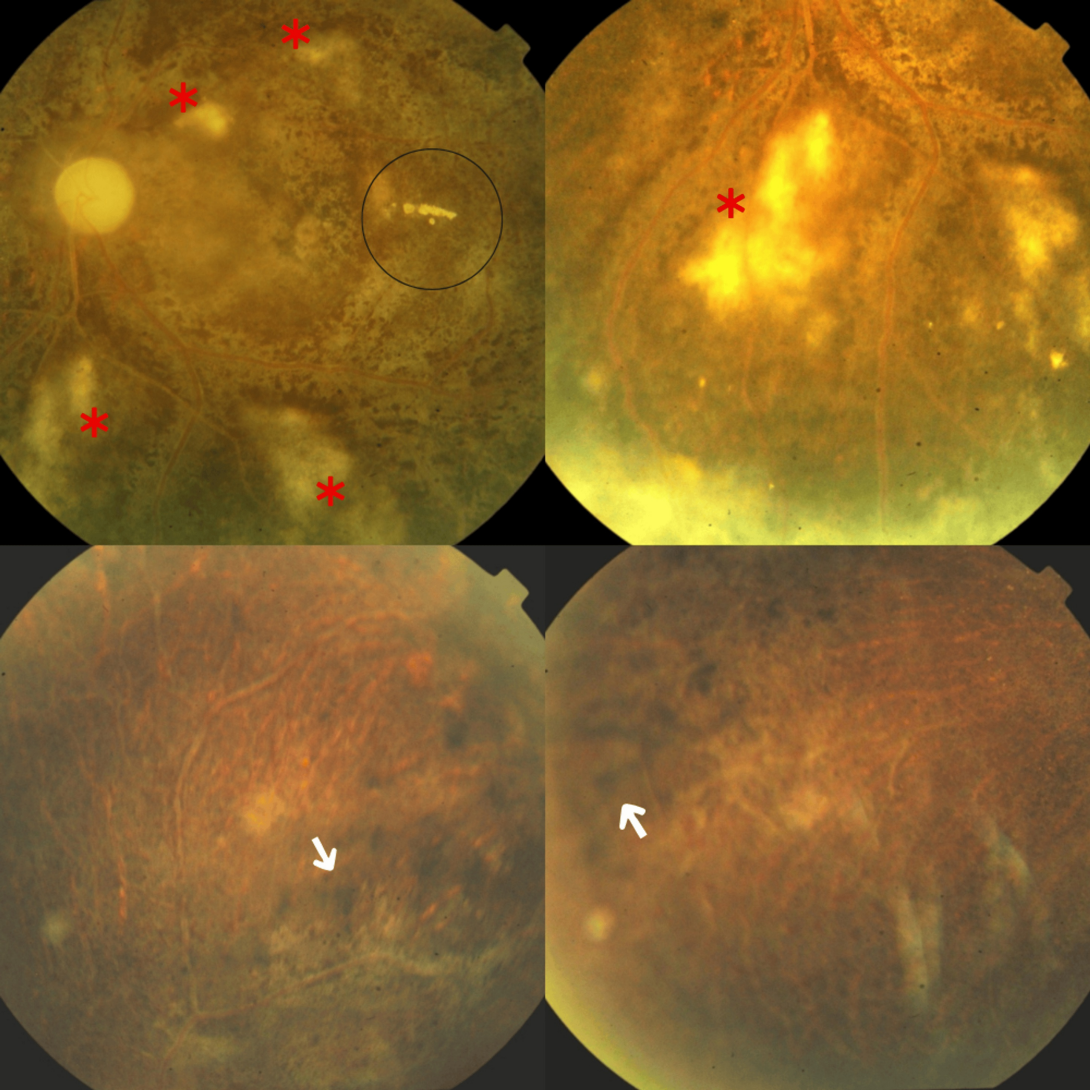
Fundus photographs of the left eye showing a pale atrophic optic disc with attenuated vasculature and vascular sheathing Few hard exudates are visible on the macula and in the peripheral retina. Additional findings include multiple ghost vessels, mottling of the retina with generalized retinal atrophy, and multiple active choroidal lesions. The black circle marks exudates in the paramacular area. The white arrow marks peripheral hyperpigmentary changes. The asterisk marks the choroidal lesions.

Fundus examination revealed bilateral vitritis (+1), pale atrophic optic discs, attenuated vessels with vascular sheathing, and multiple ghost vessels in the periphery. There was mottling of the retina with generalized retinal atrophy, along with multiple active choroidal lesions. In the right eye, sclerosed vessels were noted in the superior arcade, accompanied by a hyperpigmented patch at the macula. The left eye showed a few exudates around the macula (Figure 2, Figure 3).

**Figure 4:**
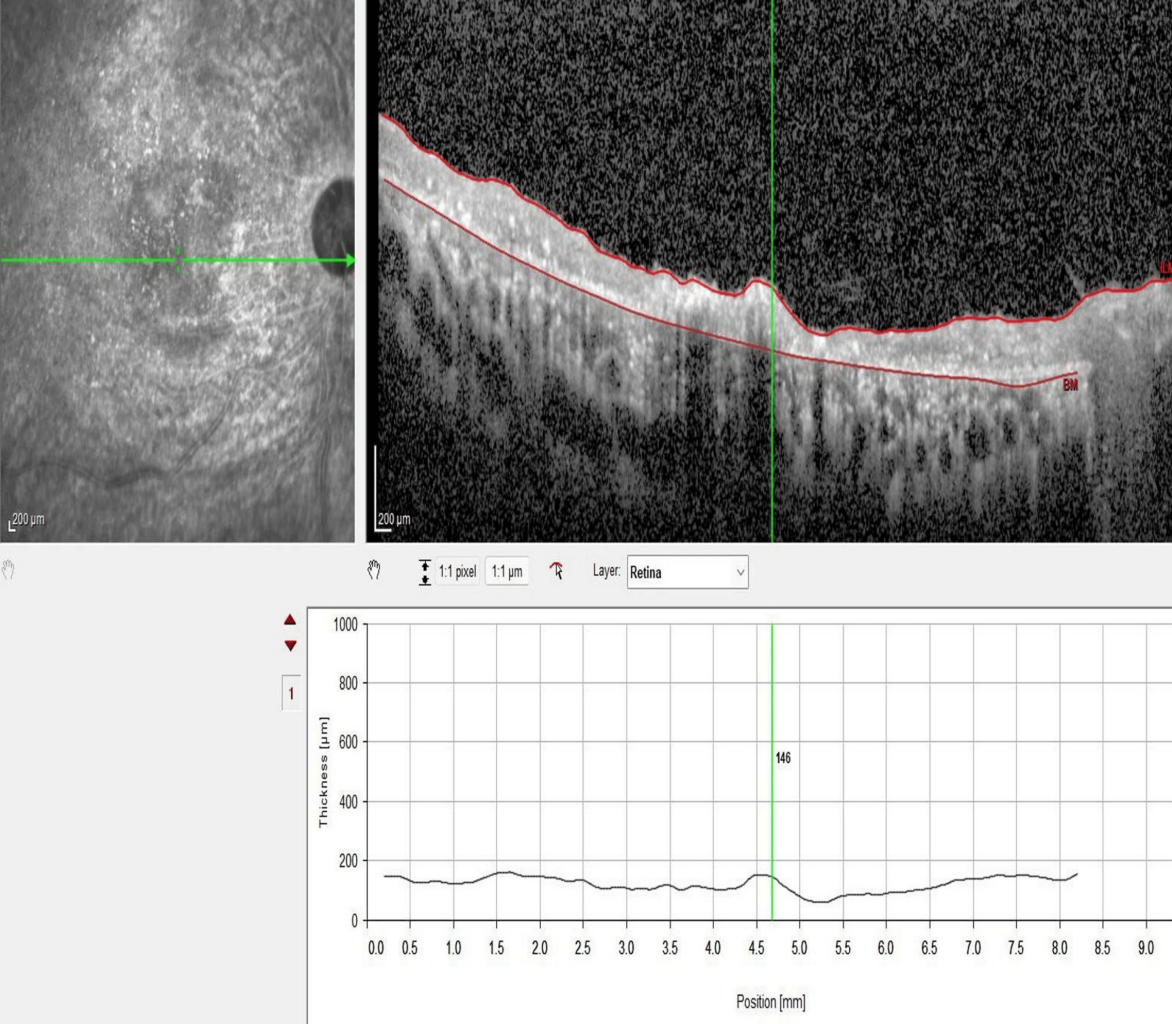
OCT of the macula and thickness profile of the right eye showing generalized retinal atrophy and a thickened choroid measuring 387 microns OCT, optical coherence tomography

**Figure 5:**
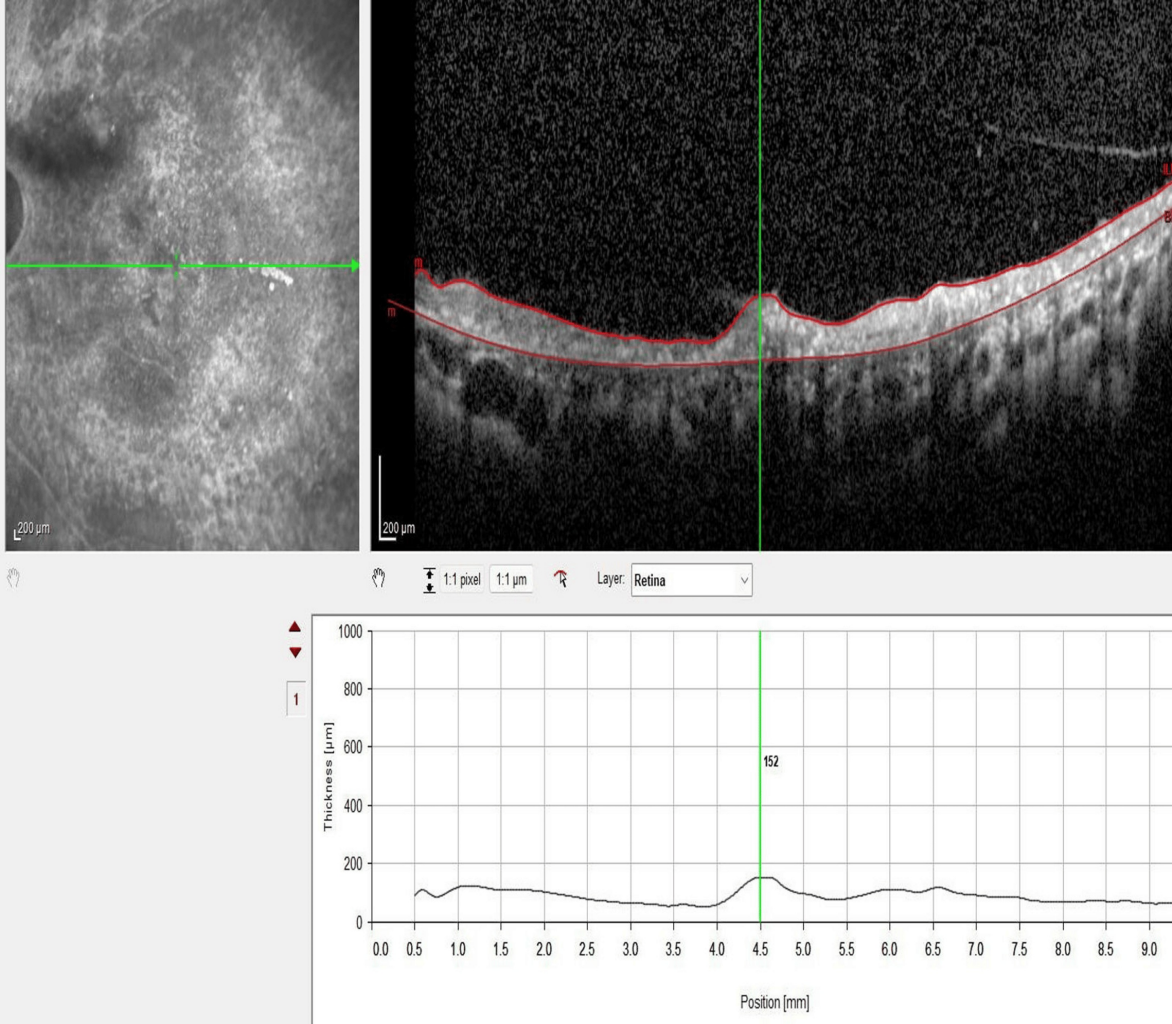
OCT of the macula and thickness profile of the left eye revealing generalized retinal atrophy, vitreomacular traction, and a thickened choroid measuring 418 microns OCT, optical coherence tomography

**Figure 6:**
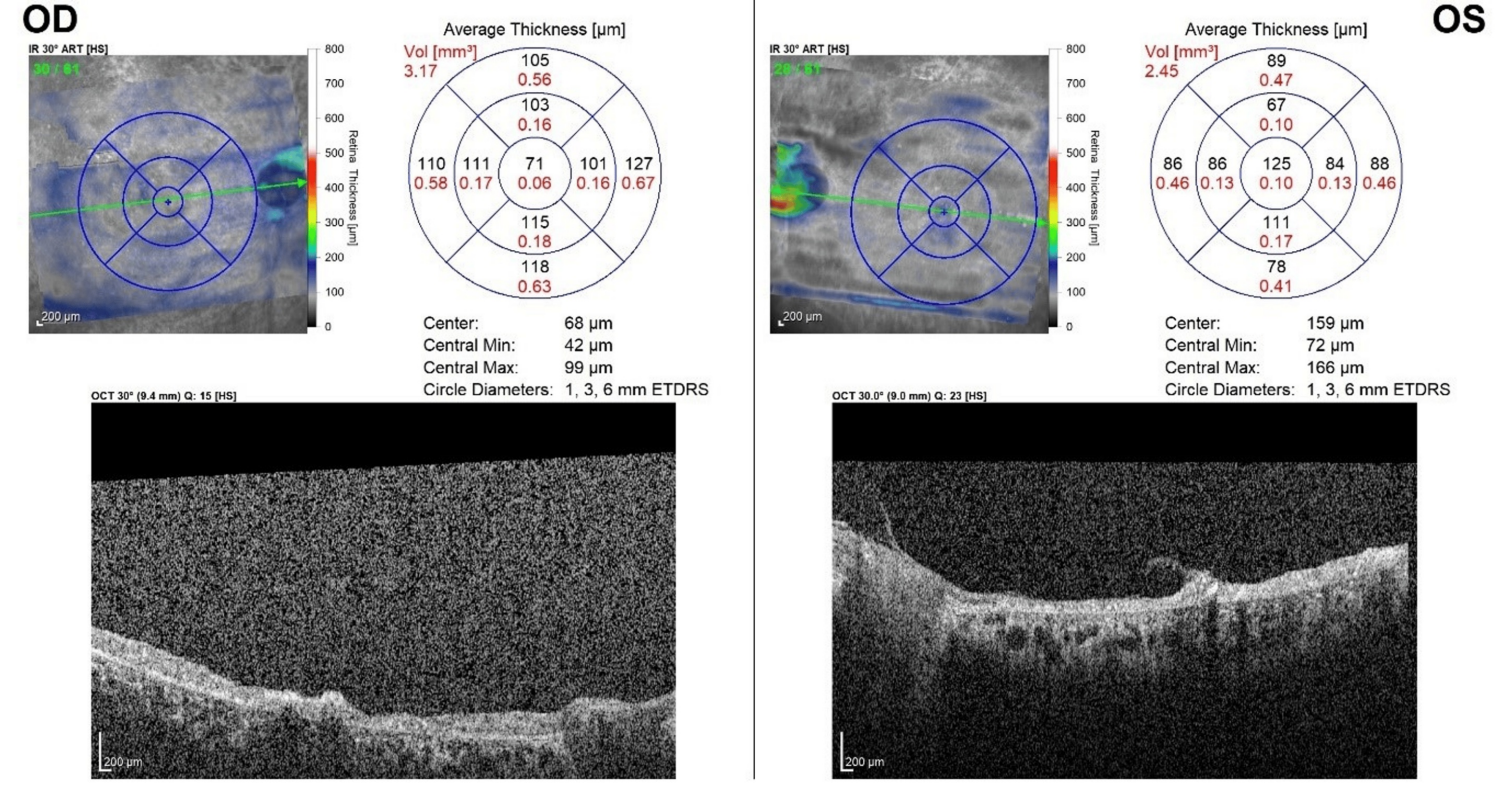
Macular thickness map of both eyes demonstrating generalized retinal atrophy The left eye shows evidence of both vitreomacular and vitreoretinal traction.

**Figure 7:**
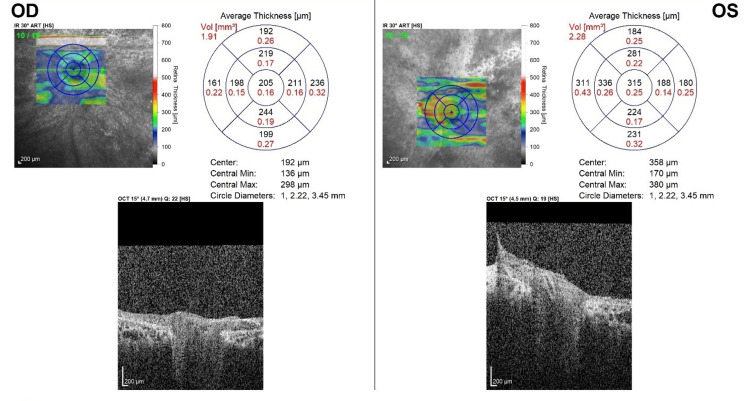
Optic nerve head thickness map of the right (OD) and left (OS) eyes showing bilateral absence of the optic cup and optic disc atrophy The left eye displays vitreoretinal traction, which results in a falsely elevated thickness measurement.

Optical coherence tomography (OCT) of the macula revealed bilateral generalized retinal atrophy with choroidal thickening. Additionally, vitreomacular and vitreoretinal traction were noted in the left eye (Figure 4, Figure 5, Figure 6). OCT retinal nerve fiber layer demonstrated absent optic disc cups and optic disc atrophy in both eyes. Notably, the vitreoretinal traction in the left eye led to a false increase in the thickness map (Figure 7).

Systemic examination revealed a child with short stature, cushingoid facies, and truncal obesity. Additional findings included dermatitis herpetiformis, oral ulcers, angular glossitis, and cheilosis.

The medication history showed that the child was initially started on oral prednisolone 15 mg once daily for 15 days, which was later reduced to 5 mg once daily, along with topical cyclopentolate 1% eye drops. However, she began developing cushingoid features, prompting a tapering of oral prednisolone and initiation of prednisolone 1% eye drops twice daily. She had also been taking over-the-counter multivitamins for one month.

Due to the severity of the disease and lack of remission despite oral corticosteroids, as well as the development of cushingoid features, steroid-sparing therapy was considered. The patient was managed by a multidisciplinary team, including ophthalmologists, a pediatrician, a nutritionist, an immunologist, and a pediatric rheumatologist. Azathioprine (25 mg/day) was initiated, topical cyclopentolate was continued, and oral prednisolone was gradually tapered.

Although the child was reportedly on a GFD, parental counseling revealed ongoing risks of cross-contamination at home through shared cookware and utensils. After a thorough dietary review, the family was counseled on strict prevention of cross-contamination, which had likely been compromising the effectiveness of the GFD.

Laboratory investigations showed sideropenic anemia and findings consistent with acute inflammation and a confirmed diagnosis of CD (Table 1). Other potential causes of uveitis - including tuberculosis, Behçet’s disease, juvenile idiopathic arthritis, sarcoidosis, toxocariasis, toxoplasmosis, ankylosing spondylitis, Kawasaki disease, systemic lupus erythematosus, Crohn’s disease, and Vogt-Koyanagi-Harada disease - were ruled out through clinical, radiological, and laboratory evaluation.

**Table 1:** Summary of the patient’s significant systemic laboratory investigations Values outside the normal range are highlighted in bold. Elevated values are indicated with a double asterisk (**), while decreased values are marked with a single asterisk (*). ACE, angiotensin-converting enzyme; AMA, anti-mitochondrial antibodies; ANA, anti-nuclear antibodies; ASMA, anti-smooth muscle antibodies; ESR, erythrocyte sedimentation rate; IGRA, interferon gamma release assay; MCH, mean corpuscular hemoglobin; MCHC, mean corpuscular hemoglobin concentration; MCV, mean corpuscular volume; RDW, red cell distribution width; RPR, rapid plasma reagin; tTg-IgG, tissue transglutaminase immunoglobulin G; VDRL, venereal disease research laboratory.

Parameter	Laboratory findings	Reference range
Hemoglobin	8.2 g/dL^*^	11.5-15.5 g/dL
Hematocrit	33.1%^*^	35.0-45.0%
MCV	55.1 fL^*^	77-95 fL
MCH	13.6 pg^*^	25-33 pg
MCHC	24.8 g/dL^*^	31-37 g/dL
RDW	20.9%^**^	12.1-16.9%
ESR	5 mm/1st hr^*^	0-20 mm/1st hr
Peripheral film	Microcytic hypochromic anemia, anisocytosis, poikilocytosis, polychromasia, target cells, and teardrop cells	-
Serum tTg-IgG	21.7 U/mL^**^	<6 U/mL
Mantoux test	<5 mm	-
IGRA gold	Negative	-
Serum ACE levels	106 U/L^**^	8-65 U/L
ANA	Negative	-
AMA	Negative	-
ASMA	Positive	-
VDRL/RPR	Nonreactive	-

The child was kept under close follow-up and maintained on a strict GFD. Although remission of disease activity was achieved after 10 weeks of initiating azathioprine and GFD, she experienced rapid visual deterioration, ultimately losing her vision over the following three months.

## Discussion

CD affects approximately 1% of the global population. Uveitis, on the other hand, is rare in children; among 20 pediatric patients with uveitis, only one was found to have CD [1,5]. CD is triggered by an immune response to prolamins and gliadin, which leads to the production of various antibodies such as anti-endomysial antibodies (EMAs) and anti-tissue transglutaminase 2 antibodies, typically in individuals with HLA-DQ2 and/or HLA-DQ8 haplotypes [1,2,4].

Although the exact mechanism linking CD and uveitis remains unclear, several hypotheses have been proposed. One of the more plausible explanations involves increased intestinal permeability to gliadin proteins, which act as antigens and stimulate lymphocyte activation, resulting in an autoimmune response [2]. The clinical presentation of CD varies and often reflects a mix of immune-mediated and genetically influenced manifestations. For instance, 25% of CD patients present with oral ulceration, either due to malabsorption-related vitamin deficiencies or a possible association with Behçet’s disease [1,2,4].

In this case, a thorough diagnostic workup revealed a clear link between CD and uveitis, with no alternative etiology identified. To our knowledge, this is the first case report from Pakistan describing bilateral posterior uveitis with spillover anterior uveitis secondary to CD in an 11-year-old girl. Although cases of this association are rare globally, existing reports consistently suggest that a GFD is the cornerstone of treatment, leading to uveitis remission (Table 2).

**Table 2:** Summary of published cases describing demographics, presenting symptoms, treatment responses, and clinical outcomes in patients diagnosed with both CD and uveitis AC: anterior chamber; CD: celiac disease; GFD: gluten-free diet; KP: keratic precipitate; N/A: Not Available

Authors	Age and gender	Signs and symptoms	GI symptoms (at presentation)	CD diagnosed after uveitis	On GFD	Response to steroids (oral/topical)	Remission with GFD
Arikan-Ayyildiz et al. [1]	11-year-old female	Height and weight <3rd percentile (growth failure); unilateral uveitis with posterior synechiae	No	Yes	Not diagnosed	Resistant	Yes
Hmidchat et al. [2]	25-year-old female	Bilateral decreased vision; AC flare; vitritis; macular edema	Yes (malabsorptive syndrome with chronic diarrhea, sideropenic anemia, dyspepsia, and weight loss)	Yes	Not diagnosed	Unable to determine (GFD and steroids started concurrently)	Yes
	11-year-old female	Unilateral pars planitis; optic disc hyperemia; macular edema	Yes	Known case	Noncompliant (resulted in decreased vision and diarrhea)	Unable to determine (GFD and steroids started concurrently)	Yes
Hyrailles et al. [3]	23-year-old male	Uveitis	N/A	Known case	Noncompliant (resulted in decreased vision)	N/A	Yes
Krifa et al. [6]	9-year-old female	Weight loss; anemia; unilateral decreased visual acuity; few small fine KPs; quiet AC; +2 vitritis (unilateral intermediate uveitis)	No	Yes	Not diagnosed	Resistant	Yes
Lachar et al. [7]	25-year-old female	Unilateral panuveitis with papillitis; serous retinal detachment; perivascular sheathing; retinal pigmentary changes	N/A	Yes	Not diagnosed	Temporary remission followed by recurrence of uveitis	N/A
	14-year-old male	Bilateral intermediate uveitis; papilledema; macular serous retinal detachment	N/A	Known case	No	Temporary remission followed by recurrence of uveitis	N/A
Klack et al. [8]	28-year-old female	Refractory uveitis	Yes	Yes	Not diagnosed	Resistant	Yes
Chiguer et al. [9]	14-year-old female	Unilateral panuveitis with papillitis and serous retinal detachment	No	Known case	Noncompliant	Resistant	Yes

Referring to Table 2, seven out of nine reported cases involved female patients, which is likely attributed to the higher prevalence of autoimmune conditions among females. In five of the nine cases, the diagnosis of CD was made after the patient presented with uveitis. For example, a case from Tunisia described a nine-year-old girl with type 1 diabetes who presented with weight loss, anemia, and unilateral intermediate uveitis. After her uveitis workup was inconclusive, an intestinal biopsy and serologic tests - including anti-endomysial and anti-transglutaminase antibodies - confirmed the diagnosis of CD. Notably, she exhibited no typical GI symptoms, and her uveitis resolved after initiating a GFD, despite being unresponsive to oral and topical steroids [6].

These findings align with a similar case involving an 11-year-old girl who presented with growth failure and unilateral uveitis. She, too, lacked GI symptoms, and her uveitis improved significantly following the start of a GFD [1]. Another case involved a 25-year-old woman who presented with unilateral panuveitis accompanied by papillitis, serous retinal detachment, perivascular sheathing, and retinal pigmentary changes. Her symptoms temporarily improved with oral and topical steroids but recurred until a GFD was introduced, leading to complete resolution [7].

In our case, the absence of KPs alongside the presence of AC cells may be attributed to early oral steroid use, which possibly reduced AC activity. Alternatively, the anterior segment inflammation could represent spillover from posterior uveitis.

A striking observation across all reported cases - whether CD was newly diagnosed following uveitis onset or whether patients with established CD and poor dietary compliance developed uveitis - is the temporary and minimal response to corticosteroid therapy (oral, topical, or intravitreal). Conversely, initiating a GFD led to a marked and sustained remission of uveitis in these patients [1-3,6-9].

Extraintestinal manifestations of CD appear to be more common than the classic GI symptoms such as bloating, diarrhea, abdominal pain, and steatorrhea [6]. Approximately half of CD patients present with extraintestinal or atypical manifestations [10]. Prior literature reports that only two out of five undiagnosed CD patients exhibited typical GI or malabsorptive symptoms [2,8]. Children with these hallmark symptoms are more likely to be diagnosed early. However, many patients lacking such symptoms only receive a diagnosis after presenting with uveitis [1,6]. Initial diagnosis relies on serologic testing, such as anti-tissue transglutaminase IgA and anti-EMA, which is then confirmed by intestinal biopsy [3,6,10].

Available evidence indicates that uveitis associated with CD is more commonly reported in female children and young women, and may present unilaterally or bilaterally. Cases include anterior, intermediate, posterior, and mixed forms of uveitis. A cohort study involving 148 patients with CD reported a hazard ratio of 1.32 and an absolute risk of 50 per 100,000 person-years for developing uveitis, even more than five years after the initial CD diagnosis [6].

Given the irreversible severe vision loss observed in our case, it is crucial to consider CD as a potential underlying cause of uveitis, particularly when common etiologies have been ruled out, even in the absence of GI symptoms.

## Conclusions

CD should be recognized as a systemic condition, and its diagnosis should be considered even in the absence of GI symptoms. A high index of suspicion for CD is warranted in cases of uveitis that are unresponsive to corticosteroid therapy. Notably, all reported cases demonstrate poor control of uveitis with topical and oral steroids. A gluten-free diet is the mainstay of management in this disease; it is known to protect duodenal mucosa, and patients not adhering to GFD are more likely to develop autoimmune manifestations of celiac disease.
